# Chronic α-Synuclein Accumulation in Rat Hippocampus Induces Lewy Bodies Formation and Specific Cognitive Impairments

**DOI:** 10.1523/ENEURO.0009-20.2020

**Published:** 2020-06-15

**Authors:** Danielle Walu Kasongo, Gioacchino de Leo, Nunzio Vicario, Giampiero Leanza, Giuseppe Legname

**Affiliations:** 1 B.R.A.I.N. Laboratory for Neurogenesis and Repair, Department of Life Sciences, University of Trieste, Trieste 34127, Italy; 2 Section of Physiology, Department of Biomedical and Biotechnological Sciences, University of Catania, Catania 95123, Italy; 3 Molecular Preclinical and Translational Imaging Research Centre - IMPRonTE, University of Catania, Catania 95125, Italy; 4 Department of Drug Sciences, University of Catania, Catania 95125, Italy; 5 Department of Neuroscience, Scuola Internazionale Superiore di Studi Avanzati (SISSA), Laboratory of Prion Biology, Trieste 34127, Italy

**Keywords:** α-synuclein, hippocampus, preformed fibrils, rat, spatial working memory, synucleinopathy

## Abstract

Occurrence of Lewy bodies (LBs)/Lewy neurites (LNs) containing misfolded fibrillar α-synuclein (α-syn) is one of the pathologic hallmarks of memory impairment-linked synucleinopathies, such as Parkinson’s disease (PD) and dementia with LBs (DLB). While it has been shown that brainstem LBs may contribute to motor symptoms, the neuropathological substrates for cognitive symptoms are still elusive. Here, recombinant mouse α-syn fibrils were bilaterally injected in the hippocampus of female Sprague Dawley rats, which underwent behavioral testing for sensorimotor and spatial learning and memory abilities. No sensorimotor deficits affecting Morris water maze task performance were observed, nor was any reference memory disturbances detectable in injected animals. By contrast, significant impairments in working memory performance became evident at 12 months postinjection. These deficits were associated to a time-dependent increase in the levels of phosphorylated α-syn at Ser129 and in the stereologically estimated numbers of proteinase K (PK)-resistant α-syn aggregates within the hippocampus. Interestingly, pathologic α-syn aggregates were found in the entorhinal cortex and, by 12 months postinjection, also in the vertical limb of the diagonal band and the piriform cortices. No pathologic α-syn deposits were found within the substantia nigra (SN), the ventral tegmental area (VTA), or the striatum, nor was any loss of dopaminergic, noradrenergic, or cholinergic neurons detected in α-syn-injected animals, compared with controls. This would suggest that the behavioral impairments seen in the α-syn-injected animals might be determined by the long-term α-syn neuropathology, rather than by neurodegeneration per se, thus leading to the onset of working memory deficits.

## Significance Statement

Cognitive deficits represent significant non-motor manifestation of Parkinson’s disease (PD) and dementia with Lewy body (LB), which deteriorates daily living activities leading to reduced independence, quality of life, and survival of affected patients. Therefore, the identification of anatomic and neuronal substrates underlying cognitive impairment can help in the development of appropriate treatments. Our data, confirm and extend previous observations showing that hippocampal α-synuclein (α-syn) pathology contribute to specific memory impairment. Thus, the α-syn preformed fibril (α-syn PFF) infusion procedure in the rat may represent a feasible tool to model synucleinopathies with which to test possible therapeutic interventions.

## Introduction

α-Synuclein (α-syn) is an intrinsically disordered 140-amino acid brain protein, predominantly α-helical structured when bound to membranes and widely present at presynaptic terminals in soluble and membrane-associated forms (Maroteaux et al., 1988). α-Syn may fold into a β-sheet-rich structure able to polymerize into highly toxic amyloid fibrils and aggregates (Eliezer et al., 2001; Uversky, 2007). Although largely unknown, α-syn is thought to be involved in synaptic vesicle release and trafficking, physiological regulation of enzymes and transporters, and neuronal apoptotic responses (Dev et al., 2003). α-Syn also constitutes the filamentous neuronal inclusions known as Lewy bodies (LBs) and Lewy neurites (LNs), which characterize a group of neurodegenerative diseases called synucleinopathies, including Parkinson’s disease (PD), dementia with LBs (DLB), and multiple system atrophy (MSA; Lee et al., 2010; McCann et al., 2014). The pathophysiological mechanisms underlying synucleinopathies are still poorly understood; however, recent findings suggest that aggregated forms of α-syn act with a prion-like mechanism (Luk et al., 2012; Masuda-Suzukake et al., 2013; Aulić et al., 2014). Over the last decades, transgenic PD models have been generated expressing moderate levels of α-syn over long periods, as it occur in the disease (Polymeropoulos et al., 1997; Singleton et al., 2003; Antony et al., 2011; Visanji et al., 2016). While valuable, however, transgenic PD models do not recapitulate other pathologic hallmarks of the disease, including nigrostriatal degeneration and motor disturbances (Blesa and Przedborski, 2014). Neurotoxic paradigms, such as intracerebral injection of 6-hydroxydopamine (6-OHDA) in rodents (Breese and Traylor, 1971) or 1-methyl-4-phenyl-1,2,3,6-tetrahydropyridine (MPTP) in non-human primates (Langston et al., 1984), have been widely used, both leading to a rapid nigrostriatal degeneration associated to robust motor deficits (Meredith et al., 2008). However, none of these models exhibit α-syn and LBs/LNs pathology (Schober, 2004; Jackson-Lewis et al., 2012). With the introduction of viral vector-based models of PD (Kirik et al., 2002), all major PD hallmarks have been obtained in rats and mice (Decressac et al., 2012; Oliveras-Salvá et al., 2013). However, the supraphysiological level of α-syn, rapid time course of the pathology (one to two months), and circumscribed (rather than widespread) α-syn aggregation seen in these animals have raised concerns (Braak et al., 2003; Oliveras-Salvá et al., 2013).

Following the hypothesis of a potential prion-like mechanism for α-syn propagation, α-syn preformed fibrils (α-syn PFFs) have been used either *in vitro* or microinjected into specific rodent brain areas *in vivo*, triggering α-syn pathology, synaptic dysfunction, perturbations in cell excitability, and cell death *in vitro* (Luk et al., 2009; Volpicelli-Daley et al., 2011), as well as α-syn inclusions resembling those found in patients, also in distally located target regions, *in vivo* (Paumier et al., 2015). The α-syn PFF model, therefore, provides a valuable tool to replicate some aspects of histopathology in PD (Patterson et al., 2019).

While brainstem LBs are thought to contribute to motor symptoms, the neural substrate for cognitive symptoms in PD remains elusive and a matter of debate. Consistent with Braak hypothesis, suggesting a caudal to rostral spread of LB/LN pathology (Braak et al., 2003), several studies have reported that cortical or limbic LBs/LNs correlate with dementia in PD (Hurtig et al., 2000; Harding and Halliday, 2001; Apaydin et al., 2002; Kövari et al., 2003; Aarsland et al., 2005; Irwin et al., 2012). Interestingly, a potential hippocampal LB involvement in cognitive impairments is further supported by significant correlations between cognitive performances of DLB patients and postmortem LB pathology in hippocampal cornu ammonis (CA)1 (Adamowicz et al., 2017). Surprisingly, however, no study to date has addressed the anatomic, molecular, and functional effects of α-syn PFF following injection in the hippocampus, a region known to be crucial for learning and memory (Squire, 1992).

Considering the above results and limitations, the present study sought to investigate the progressive pathologic alterations and spreading of synthetic α-syn fibrils bilaterally injected into the hippocampus of adult rats, up to the onset of memory impairments.

## Materials and Methods

### Expression and purification of recombinant mouse α-syn

α-Syn was prepared as described previously (Huang et al., 2005). Briefly, recombinant α-syn protein was purified from *Escherichia coli* BL21 (DE3) cells expressing mouse α-syn construct from the pET11a expression vector. *E. coli* cells were grown in minimal medium at 37°C in the presence of ampicillin (100 g/ml) until OD600 of ∼0.6, followed by induction with 0.6 mm IPTG for 5 h. The protein was extracted from periplasm by osmotic shock, followed by boiling for 20 min and ammonium sulfate precipitation. The protein was next purified by anion exchange chromatography (HiTrap Q FF column, GE Healthcare), and fractions were analyzed by SDS-PAGE. Finally, the protein was dialyzed against water, lyophilized, and stored at −80°C.

### Fibrillation of mouse α-syn

Before fibrillation, the protein was filtered (0.22-μm syringe filter), and the concentration was determined by absorbance measured at 280 nm, then the fibrillation was performed as described previously (Aulić et al., 2017). Briefly, purified mouse α-syn (1.5 mg/ml) was incubated in the presence of 100 mm NaCl and 20 mm Tris-HCl, pH 7.4. Reactions were performed in a black 96-well plate with a clear bottom (PerkinElmer), in the presence of one 3-mm glass bead (Sigma) in a final reaction volume of 200 μl. Plates were sealed and incubated in BMG FLUOstar Omega plate reader at 37°C with cycles of 50 s of shaking (400 rpm, double-orbital) and 10 s of rest. After fibrillation, the reaction mixtures were ultracentrifuged for 1 h at 100,000 × *g* (Optima Max-XP, Beckman), sonicated for 5 min (Branson 2510), and resuspended in sterile PBS, aliquoted, and stored at −80°C until use. The resulting α-syn fibril assemblies were then structurally characterized by atomic force microscopy (AFM) as previously described (Aulić et al., 2017; Extended Data Fig. 1-1).

**Figure 1.**
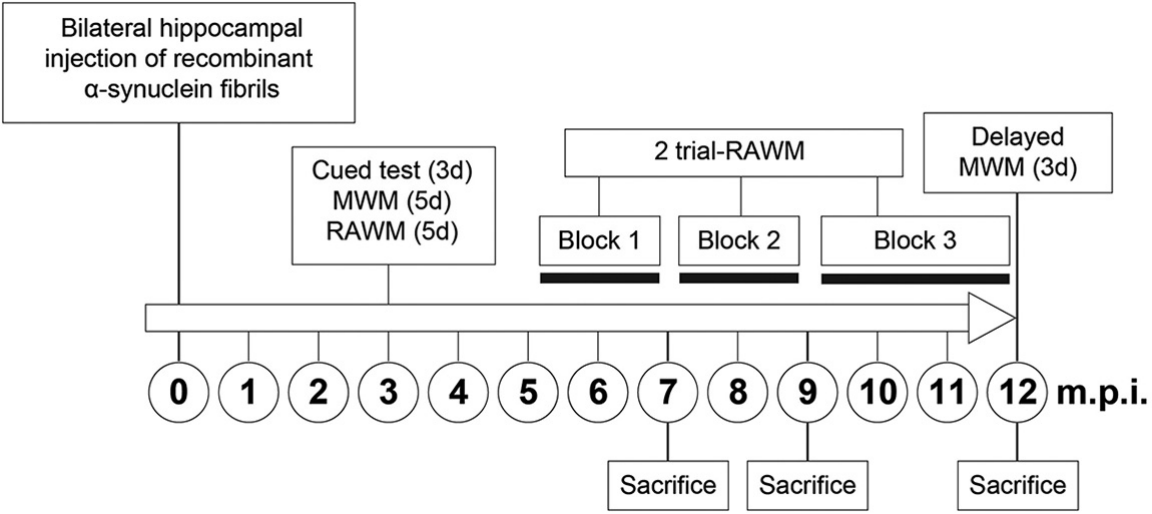
Experimental time-line diagram illustrating the temporal sequence of surgery, behavioral tests, killings, and the intervals between them. See also Extended Data Figure 1-1.

10.1523/ENEURO.0009-20.2020.f1-1 Extended Data Figure 1-1 AFM micrograph showing the assemblies of the pre-aggregated α-syn fibrils prior to their intrahippocampal inoculation. Scale bar: 1 μm. Download Figure 1-1, TIF file

### Animals and experimental design

Thirty-six young adult female Sprague Dawley rats (provided by the animal facility at the University of Trieste) weighing 220–250 g at the time of surgery were housed in high efficiency, particulate air-filtered, double-decker cage units (Tecniplast), and maintained under standard conditions of temperature, light, and humidity, with *ad libitum* access to food and water. The animals were randomly assigned to groups receiving bilateral intrahippocampal injections of recombinant mouse α-syn PFF (*n* = 18) or PBS alone (sham injected, *n* = 10), whereas the remaining animals (intact, *n* = 8) were not injected and served as unoperated controls. Subgroups of randomly selected animals (five α-syn-treated, three sham-injected, and two intact rats) were killed at approximately seven and nine months postsurgery, whereas the remaining subjects (8 α-syn-treated, four sham-injected, and four intact rats) were allowed to survive up to 12 months postinjection. Behavioral analyses were begun at approximately three months postinjection and consisted in the sequential administration of tests specifically designed to evaluate sensorimotor as well as spatial reference and working memory abilities. Upon completion of the last testing session, at each of the predetermined time points, the animals were killed, and the brains processed for quantitative immunohistochemistry or Western blot analyses. All the experimental procedures were conducted following the Italian Guidelines for Animal Care (D.L. 116/92 and 26/2014), which are in compliance with the European Communities Council Directives (2010/63/EU) and were approved by the Ethical Committee at the University of Trieste.

### Surgical procedures

Stereotaxic injections of recombinant, freshly sonicated mouse α-syn PFF bilaterally into the hippocampus were performed on rats under deep anesthesia (sodium pentobarbital, 40 mg/kg, i.p.). Briefly, 7.5 μg of the protein was injected using a 10-μl Hamilton microsyringe (Hamilton), in a volume of 5-μl sterile PBS per side at the following stereotaxic coordinates according to Paxinos and Watson (1998): AP = –3.6; ML = ±1.8; DV = –3.6 (in mm, relative to bregma and outer skull surface). The dose and volume were chosen based on the results of pilot experiments. Each infusion was conducted over 5 min, waiting for additional 2 min before withdrawal, to prevent backflow and reduce tissue damage. For sham treatment, sterile PBS was injected using the same coordinates, volume, and speed.

### Behavioral tests

Starting from approximately three months postinjection, the animals underwent a battery of behavioral tests (normally administered between 9 A.M. and 3 P.M.) conducted at different time points, to evaluate possible effects of the treatments on sensorimotor and cognitive functions and their time course (Fig. 1). All the behavioral paradigms were based on modified versions of the water maze task originally developed by Morris (1984) for the assessment of spatial learning and memory in rodents. The test apparatus consisted of a circular pool, 140 cm in diameter and 50 cm deep, filled with room temperature water to a depth of 35 cm and located in a room with several external cues that could be used for orientation. Four equally spaced points (conventionally indicated as North, South, East, and West) served as start locations, also dividing the tank into four quadrants. A circular platform (10 cm in diameter) was fixed to the bottom of the pool with it top 2 cm below (and thus invisible from) the water surface. In the middle of each quadrant, a circular area of ∼20 cm in diameter (termed annulus) indicated the site where the escape platform could have been, if placed in that quadrant. For each animal, the latency to find the hidden platform, the distance swum, and the swim speed were recorded by a computer-based video-tracking system.

Three different paradigms, designed to evaluate the sensorimotor, as well as reference and working memory abilities (Coradazzi et al., 2016; Pintus et al., 2018), were implemented.

### Cued test

All animals first received a free 60-s swim to become familiar with the swimming pool, followed by a 3-d cued learning session to exclude the occurrence of noncognitive (e.g., visual) deficits, induced by the treatment, that may interfere with the correct execution of the test. During the cued task, the position of the escape platform was made visible by a 10 × 15-cm striped flag and randomly changed on each of four daily trials.

### Morris water maze test

Three days after conclusion of the cued test, the animals were subjected to the Morris water maze test for the assessment of reference memory. The animals were given four trials per day over five consecutive days with a 30-s intertrial time. On each trial, the animal was released into the pool from one of the starting points and then given 60 s to locate the platform [constantly kept in the North-East (NE) quadrant] and climb onto it. If the submerged platform was not found, the rat was gently guided by the experimenter and left on it for ∼30 s, before be placed in the next predetermined starting point, whose sequence was defined randomly, and it was changed every day. On the fifth day of testing, after the fourth trial, the platform was removed and a spatial probe trial commenced, during which the animals were allowed to swim freely for 60 s. In this test, generally used to evaluate the efficiency of the previous learning, the distance swum as well as the collisions with the annuli in each of the four quadrants, were recorded. In order to check for possible delayed long-term effects of the α-syn treatment on reference memory, a separate 3-d Morris water maze test, with a spatial probe trial, was administered to the animals just before killing, at ∼12 months postinjection.

### Radial arm water maze (RAWM) test

Spatial working memory was tested using a RAWM apparatus consisting in six swim alleys (50 cm long × 20 cm wide, numbered 1–6) radiating out of an open central area in the same pool. The submerged escape platform was placed at the end of an arm (referred to as the goal arm), and its position was changed every day over five consecutive days. In each of the five daily trials, the animals were released from a different starting position and given up to 60 s to locate the hidden platform with a 30-s intertrial time. Entering an incorrect arm (i.e., an arm that did not contain the platform or an already visited arm) was counted as an entry error. For each trial, the latency to find the platform and the number of arm selection errors before locating the goal arm were recorded. The task design entails that the animals locate the platform by chance on the first trial each day (and thus no group difference is expected), whereas in the second and subsequent trials, the acquired information on the novel platform location for that day would determine substantial improvements in performance. Therefore, differences in latency or error scores between trials 1 and 2 across days, both in absolute terms and as percentage of trial 1 (savings) provided measures of working memory performance.

### Two-trial RAWM test

In the original five-trials-a-day paradigm, the RAWM testing is conducted over five consecutive days, allowing animals to get used to the task contingencies and to exclude all the unspecific factors that may affect performance. In this case, cutting the general duration of the test from 5 to 2 d modified the RAWM standard paradigm, as the animals were already well habituated to the environment and have learned the rules of the task. In addition, the number of trials administered every day was reduced from five to two as it was noticed previously that the main improvement in the RAWM performance mainly occurs between the first and second trial. Thus, from five months postinjection and onwards, two two-trial RAWM tests were administered over two consecutive days each week, organized as two adjacent blocks of six weekly sessions each. Starting from approximately the ninth month postinjection, the animals received the same two two-trial RAWM testing sessions, but every second week, until significant group differences in the savings for both latency and errors were detected over three consecutive testing sessions. Subsequently, the delayed 3-d Morris water maze test was administered, as mentioned above (see also Fig. 1).

### Postmortem procedures

Upon conclusion of behavioral tests, at approximately seven and nine months postinjection, the animals under terminal anesthesia (chloral hydrate, 350 mg/kg, i.p.) were perfused through the ascending aorta with room temperature saline, followed by ice-cold phosphate-buffered 4% paraformaldehyde (pH 7.4). The brains were rapidly removed, kept in the same fixative for 2 h and then transferred in a phosphate-buffered 20% sucrose solution at 4°C until they had sunk. Coronal sections at 40-μm thickness were cut using a freezing microtome (Leitz Wetzlar) from the prefrontal cortex level through the basal forebrain to the level of the caudal hippocampus and collected into six series. The sections were then stored at –20°C in a phosphate-buffered antifreeze solution containing 30% glycerol and 30% ethylene glycol, pending immunohistochemical analysis.

Animals allowed to survive up to 12 months postinjection, under terminal anesthesia as above, were perfused through the ascending aorta with room temperature saline. After rapid removal, and using a counterbalanced left–right dissection schedule, the whole hippocampus, the prefrontal, fronto-parietal, and entorhinal cortices from one hemisphere were dissected free onto a chilled glass plate, immediately frozen in crushed dry-ice, and kept at –80°C for Western blot assay (see section Western blotting). The remaining portions of the brain, which comprised the cortical and hippocampal regions from the opposite hemisphere, both striata and the entire basal forebrain were fixed by 24-h immersion in ice-cold phosphate-buffered 4% paraformaldehyde (pH 7.4), soaked in phosphate-buffered 20% sucrose and then cut as above.

### Immunohistochemistry

For the detection of proteinase K (PK) resistant α-syn aggregates, one series of free-floating coronal sections were first quenched in 3% H_2_O_2_ and 10% methanol for 10 min to eliminate endogenous peroxidase activity, and then treated 30 min with 5 μg/ml of PK. After blocking unspecific binding sites with 5% normal goat serum (NGS; Immunologic Sciences), and 0.3% Triton X-100 in PBS (KPBS, pH 7.4) for 2 h, the sections were incubated overnight with a rabbit anti-α-syn primary antibody (C20-R, Santa Cruz Biotechnology, 1:500), 2% NGS, and 0.3% Triton X-100 in KPBS. Subsequently the sections were incubated with a biotinylated secondary antibody (goat anti-rabbit 1:300, Vector), 2% NGS, and 0.3% Triton X-100 in KPBS for 1 h. After this step, the sections were incubated with avidin biotin peroxidase complex (Vectastain ABC alite kit, Vector) for 1 h and then reacted with 0.025% diaminobenzidine and 0.01% H_2_O_2_ in KPBS for 3–5 min. The sections were mounted on gelatin-coated slides, dehydrated through steps in ascending alcohol concentrations, clarified in xylene, and coverslipped for subsequent microscopical analyses. In order to ensure consistency during morphometric analyses (see below), tissue processing and staining were conducted under identical conditions, using all relevant sections at a time. Control tissue specimens, not exposed to the primary antibody, were also used to check for nonspecific labeling. All steps were performed at room temperature.

Cresyl violet staining was performed as previously described (Gulino et al., 2019). Briefly, sections were dehydrated with increasing ethanol and then soaked in xylene. Dehydrated sections were then rehydrated and stained with a solution of 0.2% sodium acetate, 1% cresyl violet, and 3% glacial acetic acid in water for 10 min at room temperature. Sections were then washed in water, dehydrated through steps in ascending alcohol concentrations, clarified in xylene, and coverslipped.

For double immunofluorescence, free-floating sections were incubated overnight at room temperature with a rabbit anti-phospho-α-syn(Ser129; D1R1R) primary antibody (Cell Signaling, 1:1000) and a monoclonal mouse anti-GFAP antibody (Sigma, 1:400). The sections were then incubated with an Alexa Fluor 488-conjugated goat anti-rabbit secondary antibody (Life Technologies, 1:200) and then with an Alexa Fluor 594-conjugated goat anti-mouse (Life Technologies, 1:200) for 2 h in the dark. The 4′,6-diamidino-2-phenylindole (DAPI; Sigma 1:200) compound was used for nuclear staining. Slides were mounted with Fluoromount Aqueous Mounting Medium (Sigma) and images were acquired using a Leica confocal microscope (Leica TCS SP2).

### Western blotting

The cortices and the hippocampi dissected for the measurement of non-phosphorylated, Ser129-phosphorylated α-syn and cellular prion protein (PrP^C^) expression levels were homogenized using a manual dounce homogenizer in a radioimmunoprecipitation assay (RIPA) buffer containing 150 mm NaCl, 0.1% Triton X-100, 0.5% sodium deoxycholate, 0.1% sodium dodecyl sulfate (SDS), 50 mm Tris-HCl (pH 8.0), 1× phosSTOP (Roche Diagnostic), and 1× protease inhibitor cocktail (Roche Diagnostic). The homogenates were centrifuged at 10,000 rpm for 10 min at 4°C and the supernatants collected and stored in aliquots at −80°C until use. Total protein content of brain homogenates was measured using bicinchoninic acid protein (BCA) quantification kit (Pierce) and 25 μg/ml of brain homogenates were resuspended in Laemmli loading buffer, boiled for 5 min at 100°C for denaturation. Subsequently the samples were loaded onto the Mini-PROTEAN TGX Precast Gels (Bio-Rad), transferred to PVDF membranes (Immobilon-P, Millipore), and incubated for 30 min with 4% paraformaldehyde (PFA) in PBS to better detect endogenous Ser129-phosphoryated α-syn (Sasaki et al., 2015). By contrast, no PFA treatment was employed for the detection of non-phosphorylated α-syn and PrP^C^. After incubation, the membrane was washed in TBS-T (0.1% Tween 20 in TBS) for 10 min, treated with 5% non-fat milk or bovine serum albumin (BSA; w/v, in PBS) blocking solution for 1 h at room temperature in agitation and then incubated overnight at 4°C with the following primary antibodies: rabbit polyclonal anti-phospho-S129 α-syn (Abcam, ab59264 1:1000), rabbit polyclonal anti-α-syn (C-20-R, Santa Cruz Biotechnology, 1:1000), mouse monoclonal anti-PrP^C^ (clone P, kindly provided by Prof. S. Prusiner, 1:1000), and a mouse monoclonal anti β-actin (1:50,000, A3854 Sigma-Aldrich), all diluted in blocking solution.

Membranes were washed with TBST and incubated in horseradish peroxidase (HRP)-conjugated goat anti-rabbit (DAKO, 1:1000) or goat anti-mouse (DAKO 1:1000) secondary Ab for 1 h. The membranes were washed in TBST, and proteins were visualized following the manufacturers’ instructions using GE Healthcare ECL Western Blotting Detection reagent (GE Healthcare) with UVITEC Cambridge. Quantitative densitometry analysis of proteins was performed using UVBand software (UVITEC Cambridge).

### Stereology

All analyses were conducted on coded slides by investigators blinded to the groups’ identity. The occurrence of PK-resistant α-syn-immunoreactive deposits, as either LB-like inclusions (defined as dense, darkly stained intraneuronal cores) or LN-like inclusions (defined as dense, darkly stained neurites) in the hippocampal formation, was quantified using an unbiased stereological estimation method based on the optical fractionator principle (West et al., 1991). α-Syn-immunoreactive neurons and processes were counted bilaterally in the CA1 subfield of the hippocampus and in the dentate gyrus (DG; i.e., the areas in close proximity to the injection site), using ∼10 sections per rat located between 2.1 and 4.5 mm caudal to bregma. The same analyses were also conducted to quantitatively estimate the presence of α-syn-immunoreactive deposits in projection areas outside the injection site, namely the entorhinal cortex. The sampling system consisted of an Olympus BH2 microscope (fitted with an *X*-*Y* motorized stage and a microcator to measure distances in the *z*-axis) interfaced with a color video camera (Sony) and a personal computer. The CAST GRID software (Olympus Denmark A/S) was used to delineate the hippocampus area at 4× magnification, as well as to generate unbiased counting frames which were moved randomly and systematically until the entire delineated area was sampled. Using a 100× oil objective, unambiguously positive aggregates were identified and counted. For each animal, estimates of the total numbers of neuronal and neuritic aggregates in the hippocampus and entorhinal cortex were obtained according to the optical fractionator formula and then plotted as mean ± SEM for each time point analyzed. Accuracy of the stereological procedure was evaluated following (Gundersen and Jensen, 1987), and values <0.1 were considered acceptable.

### RNA extraction and quantitative real-time PCR (qRT-PCR) analysis

Total RNA was isolated from ∼300 mg of prefrontal cortex and hippocampus from frozen postmortem rat brain tissue. RNA processing and qRT-PCR experiment were the same as reported previously (Vanni et al., 2017) and the primers sequences were as follows: for *Actb* forward CTGTGTGGATTGGTGGCTCT reverse CAGCTCAGTAACAGTCCGCC; for *Snca* forward *TGTCAAGAAGGACCAGATGGG* reverse TAGTCTTGGTAGCCTTCCTCT and for *Prnp* forward CGGTACCAGTCCGGTTTAGG reverse GCTTTTTGCAGAGGCCAACA. Differential gene expression of *Scna* and *Prnp* was normalized to *Actb* expression. The relative expression ratio was calculated using the ΔΔCT method (Livak and Schmittgen, 2001).

### Statistical analysis

Data fulfilled the criteria for normal distribution and were therefore analyzed using parametric tests for all statistical comparisons. Group differences in behavioral performance as well as in the numbers of immunostained profiles or α-syn expression levels were evaluated by either repeated or one-way ANOVA, as appropriate, followed by Fisher’s protected least significant difference (PLSD) *post hoc* test. All data are presented as mean ± SEM, and differences were considered significant at *p* < 0.05.

## Results

### Behavioral analyses

**Figure 2.**
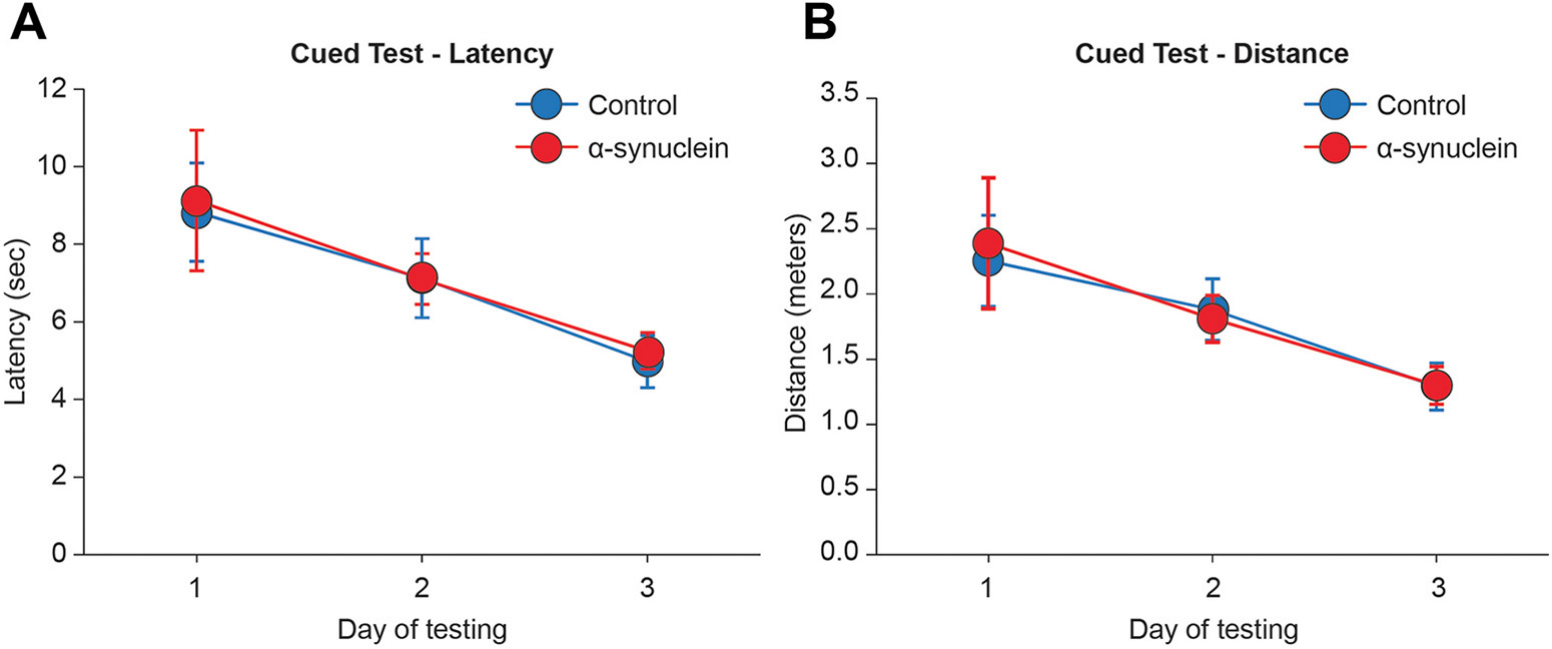
Groups performance during the cued version of the Morris water maze test at three months postinjection. In this task, the visually cued escape platform was moved to a different quadrant on each of the four daily trials, and average escape latencies (***A***) and swim distances (***B***) were recorded. Each point represents the mean value ± SEM for the block of four trials administered each day, over the three training days.

All animals, regardless of their treatment, increased in body weight and exhibited fairly normal sensorimotor functioning when evaluated in the 3-d cued test at three months postinjection (Fig. 2). In fact, the groups improved their performance over time (repeated measures ANOVA, effect of day on latency, *F*_(2,66)_ = 51.79; on distance, *F*_(2,66)_ = 42.44; both *p* < 0.001) and did not differ from each other (main group effect on latency *F*_(2,33)_ = 0.11; on distance *F*_(2,33)_ = 0.13; group × day on latency *F*_(4,66)_ = 0.23; on distance *F*_(4,66)_ = 0.23; all not significant (n.s.)). Moreover, swim speed, monitored as a measure of motor ability throughout the execution of the various tasks, averaged 0.2–0.3 m/s, and did not differ between groups at any time point, indicating that the treatments did not produce any sensory or motor impairments that would affect navigation search in the pool. As revealed by statistics, rats with sham injections did not differ from the intact animals on any of the behavioral or morphologic parameters analyzed. These animals were therefore combined into a single control group (*n* = 18) for all illustrations.

### Morris water maze test

**Figure 3.**
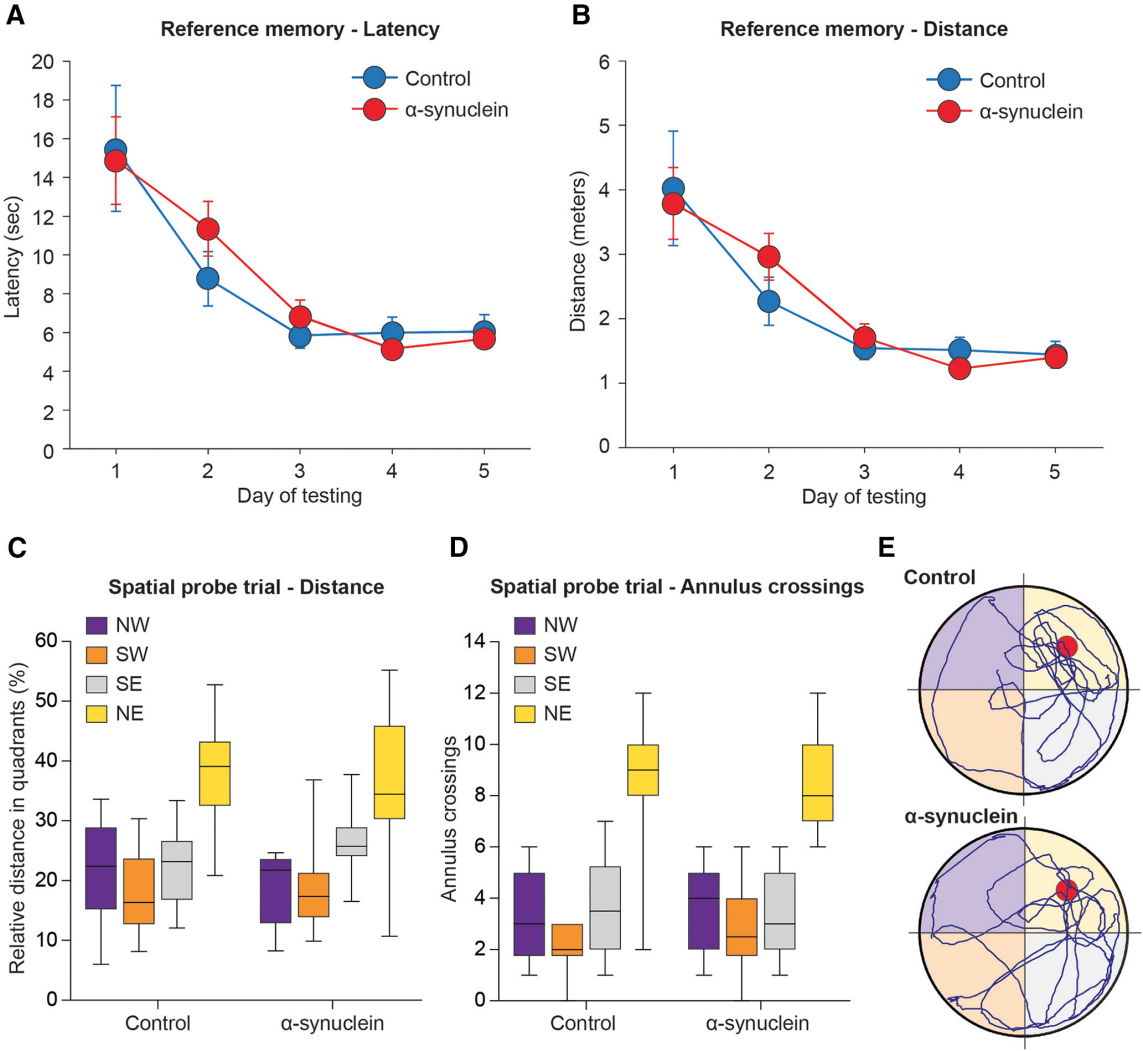
Performance during the Water maze reference memory test at three months postinjection. Average latency (***A***) and swim distance (***B***) required to locate the submerged platform during the acquisition phase of the spatial navigation task. Each sample point represents the mean value ± SEM for the block of four trials on each of the five consecutive days of testing. Lower diagrams illustrate the mean relative distance swum (***C***) and the average number of annulus crossings (***D***) in each quadrant during the spatial probe trial, once the escape platform was removed from the NE (training) quadrant. In ***E***, the actual swim paths taken by representative animals from the different groups are shown. All groups exhibited an equally efficient performance, indicating that the α-syn injection had no effects on this task. NE, North-East; NW, North-West; SE, South-East; SW, South-West.

Mean latencies and swim distances required to find the hidden platform in the reference memory version of the Morris water maze task administered at three months postinjection are shown in Figure 3*A*,*B*. All animals rapidly learned to locate the platform and improved significantly over the five testing days (repeated measures ANOVA, effect of day on latency, *F*_(4,132)_ = 43.93; on distance, *F*_(4,132)_ = 40.58; both *p* < 0.001), reaching an asymptotic performance already on day three. The animals appeared to learn the tasks at similar rates, and no significant differences were observed between the groups (main group effect on latency *F*_(2,33)_ = 0.47; on distance *F*_(2,33)_ = 0.60; group per day on latency, *F*_(8,132)_ = 0.16; on distance *F*_(8,132)_ = 0.18; all n.s.). During the spatial probe trial, on the last day of testing, when the platform was removed for a 60-s free swim (Fig. 3*C–E*), all animals swam primarily in the training (NE) quadrant, where the platform was originally located (repeated measures ANOVA, effect of quadrant on swim distance, *F*_(3,99)_ = 36.73; on annulus crossings, *F*_(3,99)_ = 74.06, both *p* < 0.001), with equal efficiency (main group effect on distance *F*_(2,33)_ = 0.67; on annulus crossings, *F*_(2,33)_ = 1.87; group per quadrant interaction for distance, *F*_(6,99)_ = 1.35; for annulus crossings *F*_(6,99)_ = 1.39; all n.s.). In addition, no group difference was observed in the total number of collisions with the annuli or swim speed (one-way ANOVA, main group effect, respectively, *F*_(2,33)_ = 1.87 and *F*_(2,33)_ = 0.01; both n.s.), indicating an equally active and spatially focused search behavior in all animals.

**Figure 4.**
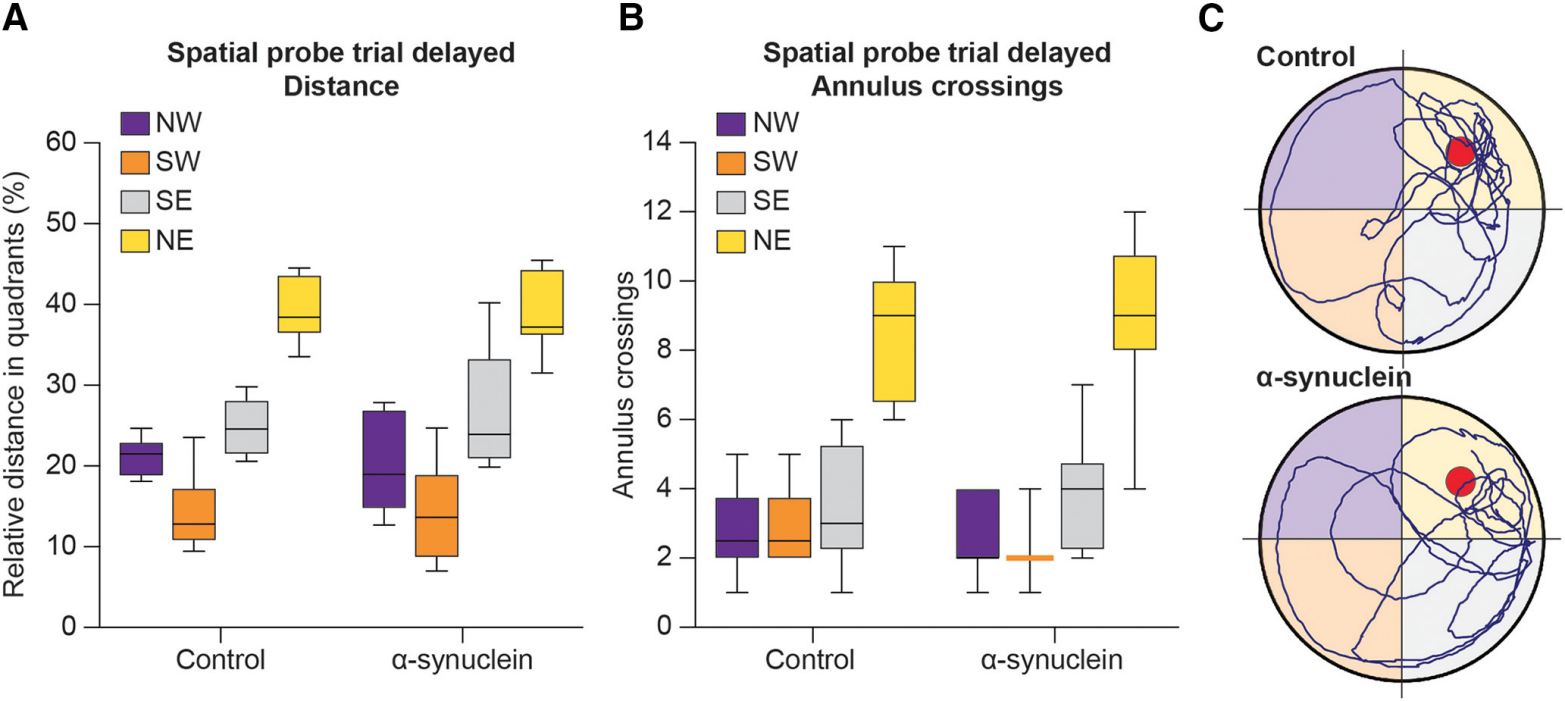
Performance during the spatial probe trial of the separate 3-d Morris water maze test administered just before killing, at 12 months postinjection. The mean relative distance swum (***A***), the average number of annulus crossings (***B***), and the actual swim paths taken by representative animals from the different groups (***C***) are illustrated. Consistent with the observations at the three-month time point, no long-term impairments in reference memory abilities were produced by the α-syn treatment.

At ∼12 months postsurgery, on conclusion of the last two-trial RAWM session (see below), the animals were tested again in the Morris water maze task to evaluate possible delayed effects of the injected α-syn PFF, as a result of their progressive spreading. Only the data referring to the spatial probe trial will be reported here (Fig. 4*A–C*). Consistent with the observations at the three-month time point, the α-syn treatment produced no long-term impairments in reference memory abilities. In fact, all tested animals exhibited an equally efficient and focused search behavior in the spatial probe trial (repeated measures ANOVA effect of quadrant on swim distance, *F*_(3.39)_ = 9.31; on annulus crossings *F*_(3.39)_ = 19.49; both *p* < 0.001), with no obvious group differences (main group effect on distance, *F*_(2,13)_ = 1.29; on annulus crossings, *F*_(2,13)_ = 2.00; group × quadrant interaction for distance, *F*_(6,39)_ = 1.25; for annulus crossings *F*_(6,39)_ = 1.76; all n.s.). This applied also to the total number of crossings or swim speed.

### RAWM test

**Figure 5.**
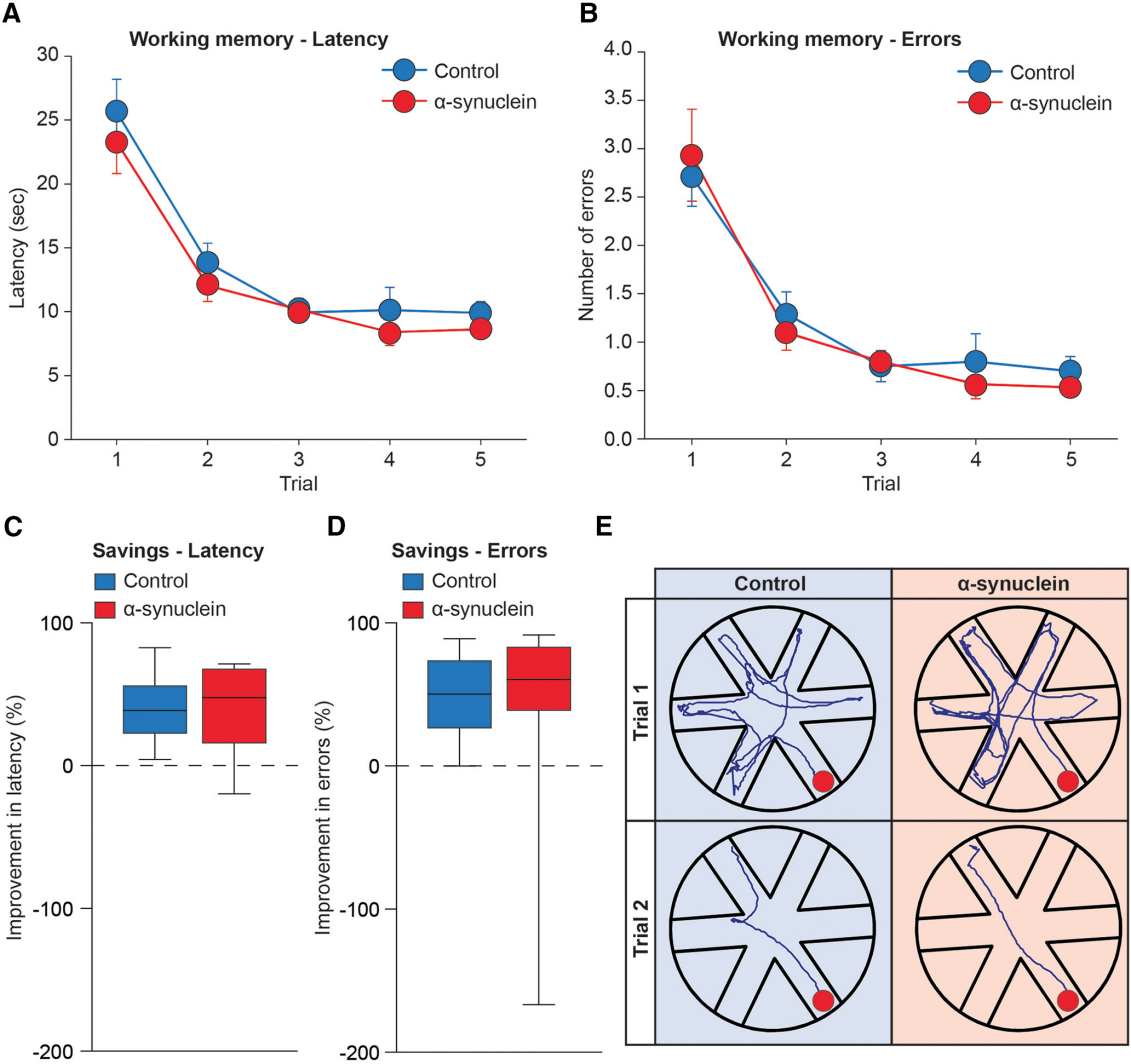
Working memory performance in the RAWM task, at three months postinjection, illustrating latency (***A***) and number of entry errors (***B***) required by the animals to find the hidden platform. Each sample point represents the mean latency and errors ± SEM recorded during each 60-s trial over five consecutive testing days. In the lower diagrams, performances are plotted as percent savings between trials 1 and 2 for latency (***C***) and errors (***D***). In ***E***, the actual swim paths taken by representative animals from the different groups are illustrated. All groups exhibited an equally efficient performance, indicating no clear-cut effects of α-syn on working memory at this time point.

Group performances in the RAWM task, when first administered at three months postinjection, are shown in Figure 5*A–E*. In this design, the platform position was moved to a new arm daily, thus the animals had to re-learn its position within the five trials of each testing day by developing a new search strategy. As expected, all animals required a longer latency and made a higher number of arm selection errors (i.e., entering an arm not containing the platform or an arm which had already been visited) during the first trial of each day, but they improved over the five trials (repeated measures ANOVA, effect of trial on latency, *F*_(4,132)_ = 57.76; on errors, *F*_(4,132)_ = 45.69; both *p* < 0.001). At the three-month time point, the ability to progressively reduce the latency and errors required to locate the hidden platform was similar for all animals, and no group difference was detected (main group effect on latency, *F*_(2,33)_ = 0.19; on errors *F*_(2,33)_ = 0.45; group per trial interaction on latency *F*_(8,132)_ = 0.67; on errors, *F*_(8,132)_ = 0.55; all n.s.). Inspection of latency and errors savings, calculated as percentage improvement between trials 1 and 2, confirmed a ∼ 40–48% reduction of latency and entry errors, with no obvious group difference (one-way ANOVA with Fisher’s PLSD *post hoc* test; all n.s.). The swim paths obtained on the fifth day of training from representative animals are shown in Figure 5*E*.

### Two-trials RAWM test

**Figure 6.**
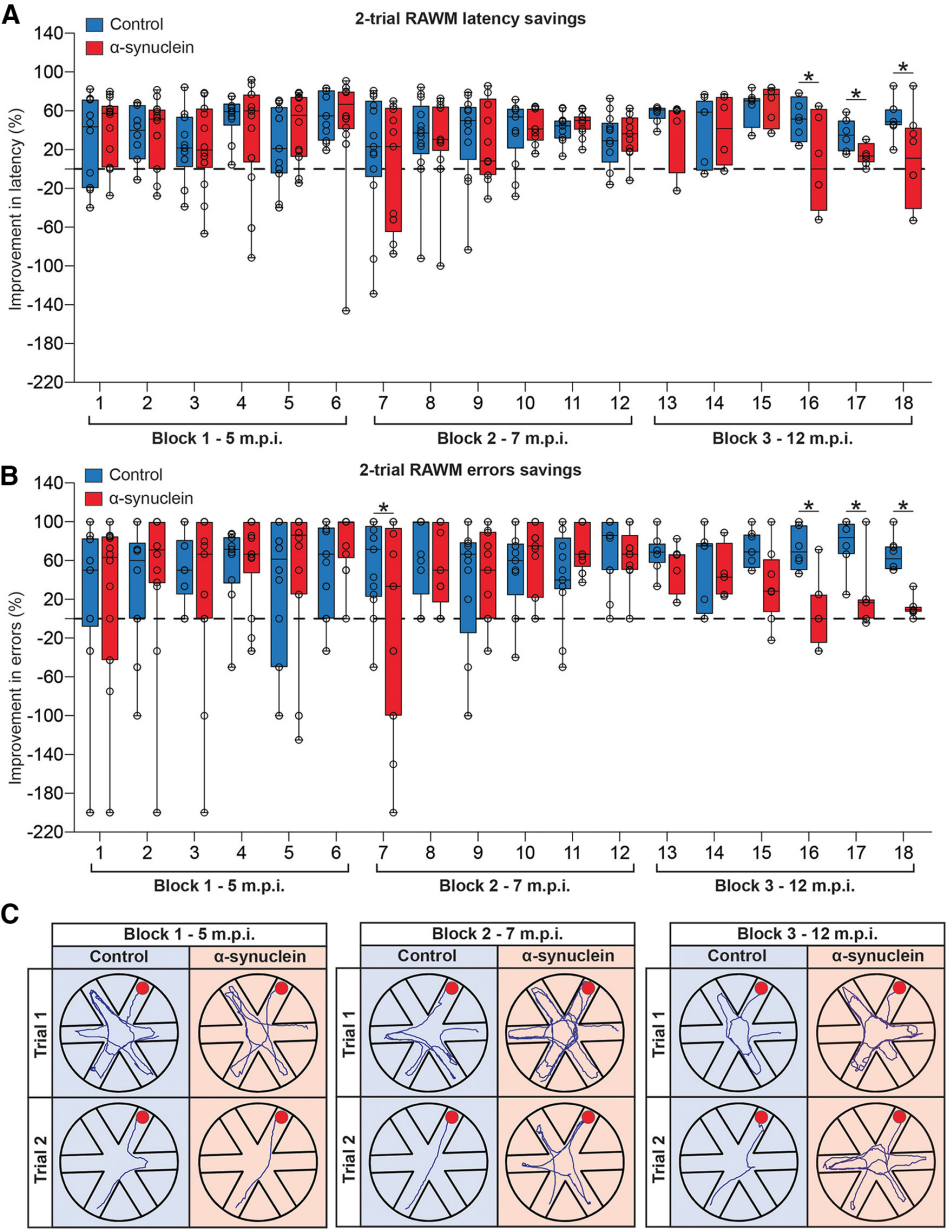
Working memory performance during the two trials RAWM, from five to 12 months postinjection. Data represent the percentage rate of improvement between the first and the second trial (savings) in latency (***A***) and errors (***B***). All groups exhibited an equally efficient working memory during the first two blocks of weekly sessions, administered at five and seven months postinjection, respectively; however, α-syn-treated animals progressively worsened their performance and appeared significantly impaired during the third block of sessions, administered every two weeks. In ***C***, the actual swim paths taken by representative animals from the different groups in the various blocks are illustrated. Asterisks indicate significant difference from control at *p* < 0.01.

Starting from five months and up to ∼12 months postinjection, the animals were tested using a modified version of the RAWM task, with two daily trials administered over two consecutive days. The testing was then organized, with intervening killings of randomly selected subjects from the various groups at different time points postinjection, so as to have three main blocks of six sessions administered weekly or every two weeks, as outlined above. By such design, latency and error savings, calculated as percentage improvement from trial 1 to 2, provided a measure of working memory performance and its possible changes over time (Fig. 6*A–C*). During the first two blocks (weekly sessions 1–6 and 7–12 starting at five and seven months postinjection, respectively) animals in the groups were equally efficient in reducing both escape latency and number of errors across trials, and no significant group difference was detected (one-way ANOVA, all *p* > 0.05, n.s.). By contrast, in the third block of sessions (administered every two weeks starting from the ninth month postinjection), α-syn-treated animals were seen to progressively worsen their performance. In fact, the calculated percent improvements in both latency and errors appeared significantly lower than those exhibited by the sham-injected and intact groups already on session 15 (one-way ANOVA, *p* < 0.01 or *p* < 0.05 for both measures), and remained unmodified up to session 18, when the training criterion (i.e., significant group differences in at least three consecutive sessions) was considered fulfilled and the two-trial RAWM testing was interrupted (actual swim paths from representative animals are illustrated in Fig. 6*C*).

### Morphologic analyses

Consistent with previous observations (Aulić et al., 2017), sonication of α-syn fibrils resulted in the formation of relatively small and homogeneous fibrillary assemblies, as revealed by AFM (Extended Data Fig. 1-1). By contrast, no such pattern was exhibited by the non-preaggregated protein (data not shown).

**Figure 7.**
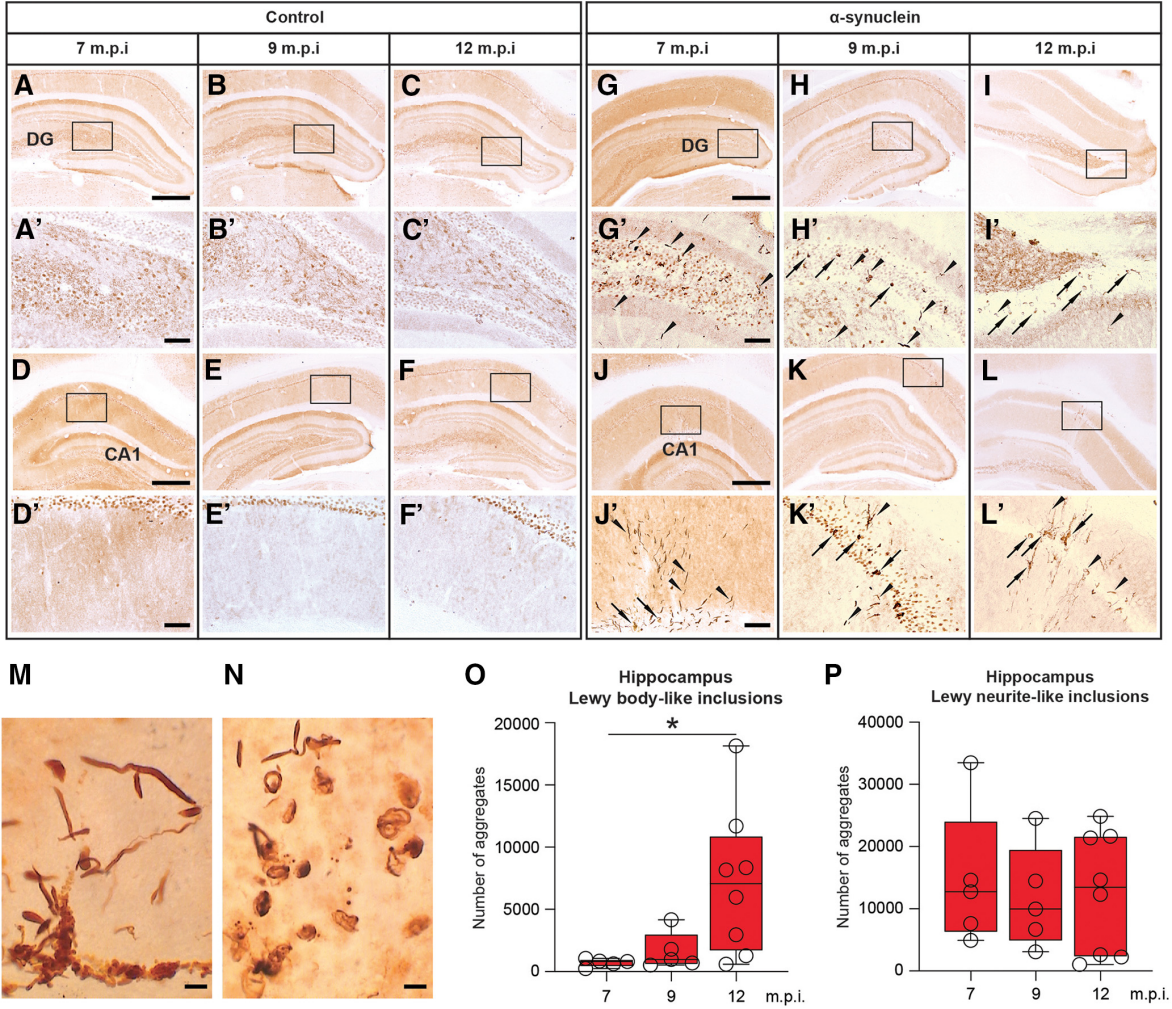
Representative micrographs illustrating, on the coronal plane, the negligible expression of PK-resistant α-syn immunoreactivity at seven, nine, and 12 months postinjection in the hippocampal DG (***A*–*C***, insets shown in ***A’–C’***) and in the CA1 region (***D–F***, insets shown in ***D’–F’***) of control rats. Progressive aggregation of α-syn after injection of recombinant mouse α-syn fibrils into the hippocampus. Compared with control, a strong PK-resistant α-syn immunoreactivity was found at seven, nine, and 12 months postinjection in the DG (***G–I***, insets shown in ***G’–I’***) and in the CA1 region (***J–L***, insets shown in ***J’–L’***) of injected rats. PK-resistant α-syn aggregates occurred as darkly stained LB-like (arrows) and LN-like deposits (arrowheads) and are shown at higher magnification in ***M***, ***N***. Box-whiskers plots illustrate the stereologically estimated numbers at different time points ± min to max value of LB-like (***O***) and LN-like (***P***) inclusions in the hippocampus of rats injected with the α-syn fibrils. Scale bars: 500 μm (in ***A***, ***D***, ***G***, ***J*** for ***A–C***, ***D–F***, ***G–I***, ***J–L***), 100 μm (in ***A’***, ***D’***, ***G’***, ***J’*** for ***A’–C’***, ***D’–F’***, ***G’–I’***, ***J’–L’***), and 5 μm (***M***, ***N***). Asterisk in ***O*** indicates significant time-dependent changes at *p* < 0.05.

**Figure 8.**
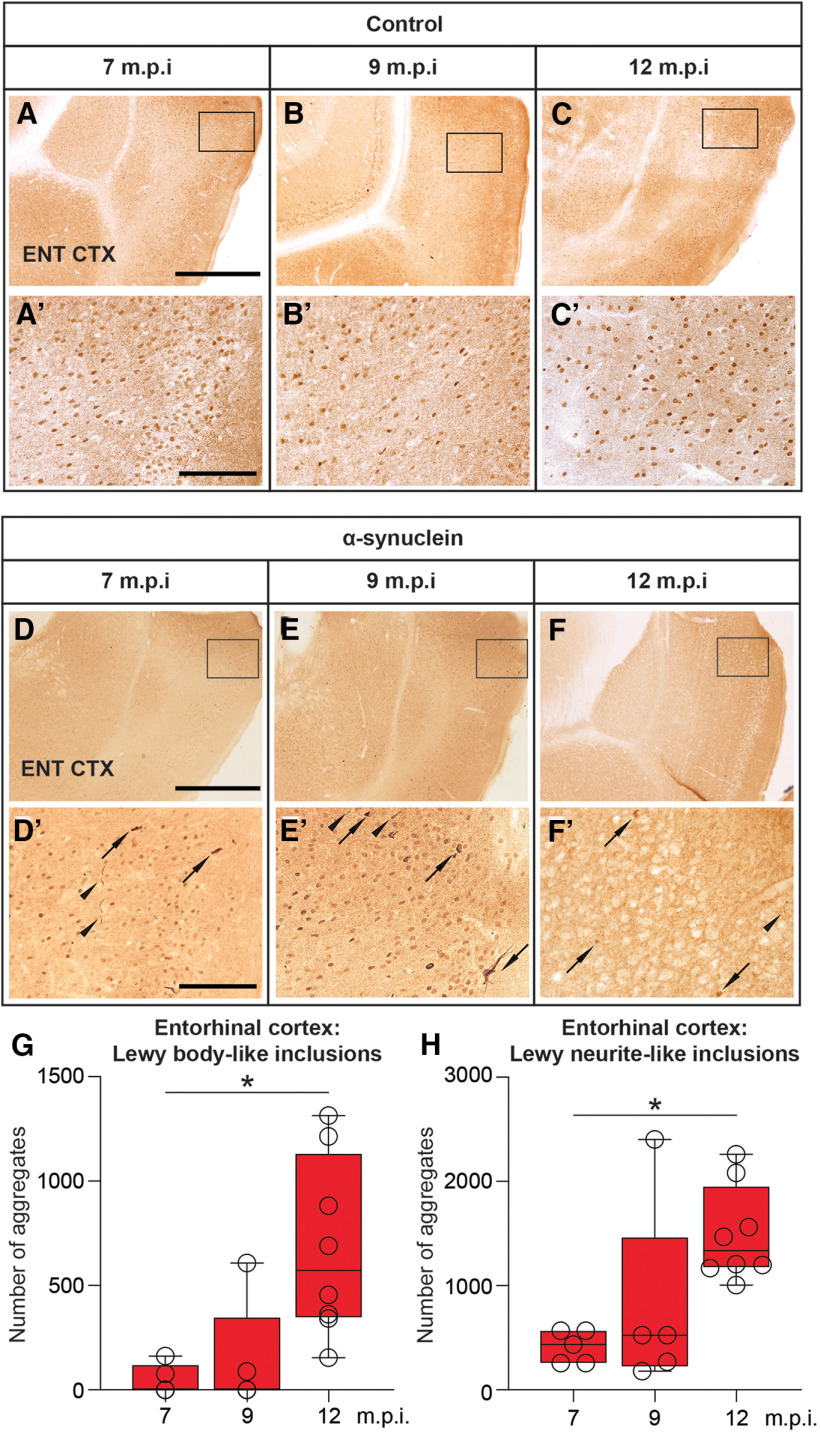
Representative micrographs illustrating, on the coronal plane, the negligible expression of PK-resistant α-syn immunoreactivity at seven, nine, and 12 months postinjection in the entorhinal cortex (***A–C***, insets shown in ***A’–C’***) of control rats. Progressive α-syn aggregation after injection of recombinant mouse α-syn fibrils into the hippocampus. A strong PK-resistant α-syn immunoreactivity occurring as both LB-like (arrows) and LN-like (arrowheads) deposits was also found at seven, nine, and 12 months postinjection in the entorhinal cortex (***D–F***, insets shown in ***D′–F′***) of injected rats. Box-whiskers plots illustrate the stereologically estimated numbers at different time points ± min to max value of LB-like (***G***) and LN-like (***H***) inclusions in the two entorhinal cortex of the injected rats. Scale bar: 500 μm (in ***A***, ***D*** for ***A–C***, ***D–F***) and 100 μm (in ***A’***, ***D’*** for ***A’–C’***, ***D’–F’***). Asterisks in ***G***, ***H*** indicate significant time-dependent changes at *p* < 0.05.

**Figure 9.**
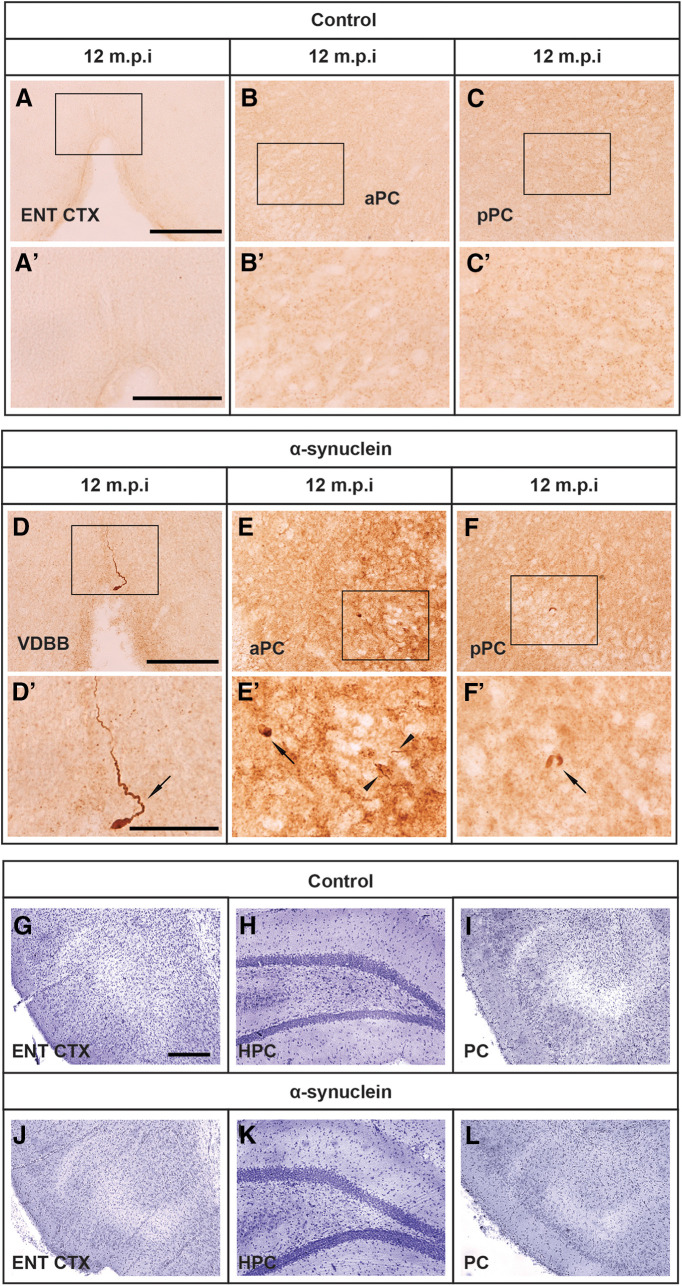
Representative micrographs illustrating, on the coronal plane, the negligible expression of PK-resistant α-syn immunoreactivity at seven, nine, and 12 months postinjection in the vertical limb of the DBB (vDBB; ***A***, inset shown in ***A′***), and the anterior and posterior piriform cortices (aPC and pPC; ***B***, ***C***, respectively, insets shown in ***B′***, ***C′***). Spreading of α-syn aggregates. A strong PK-resistant α-syn immunoreactivity was also found at 12 months postinjection in the vDBB (***D***, inset shown in ***D′***, arrow) and the anterior and posterior piriform cortices (aPC and pPC, ***E***, ***F,*** insets shown in ***E′***, ***F′***; arrows and arrowheads indicate LB-like and LN-like inclusions, respectively). Scale bar: 100 μm (in ***A***, ***D***, for ***A–C***, ***D–F***) and 50 μm (***D*** for ***D–F***). Cresyl violet staining of the entorhinal cortex (ENT CTX), hippocampus (HPC), and piriform cortices (PC) of control (***G***, ***H***, ***I***, respectively) and α-syn-injected animals (***J***, ***K***, ***L***, respectively). Scale bar: 100 μm (in ***G*** for ***G–L***).

The bilateral injection of synthetic mouse α-syn fibrils into the hippocampus resulted in the occurrence and progressive accumulation of α-syn-immunoreactive aggregates in various hippocampal subfields (e.g., DG and CA1; Fig. 7*G–L’*), whereas no such α-syn aggregates were found in the brain of sham-injected or intact animals (compare, e.g., with corresponding regions in control animals; Fig. 7*A–F’*). In general, the strong PK-resistant α-syn immunoreactivity occurred as both LB-like and LN-like deposits (Fig. 7*M*,*N*) and was detected not only in the proximity of the injection site but also in distinct anatomically related regions, with a morphology and distribution pattern that varied in a time-dependent fashion. Thus, as estimated by stereology, relatively few α-syn-immunoreactive LB-like inclusions were found in the hippocampal CA1 and DG subfields at approximately seven months postinjection, their numbers showing a significant 2.2-fold and 5.8-fold increase at nine and 12 months, respectively (one-way ANOVA with Fisher’s PLSD *post hoc* test, *p* < 0.05; Fig. 7*O*). Conversely, the number of LN-like deposits in the same regions was slightly higher at seven months postinjection and exhibited a non-significant reduction at nine and 12 months (Fig. 7*P*). LB-like and LN-like inclusions in the entorhinal cortex were virtually undetectable or very sparse at seven months postinjection, but they became clearly visible already at nine months (Fig. 8*D–F’*; compare with corresponding regions in control animals, Fig. 8*A–C’*), and their numbers increased significantly over time (one-way ANOVA with Fisher’s PLSD *post hoc* test, both *p* < 0.05; Fig. 8*G*,*H*). By contrast, only at 12 months were α-syn-immunoreactive LB-like and LN-like inclusions found in other projection areas, such as the vertical limb of the diagonal band, and the anterior and posterior piriform cortices, being barely detectable at earlier time points (Fig. 9*D–F’*; compare with corresponding regions in control animals, Fig. 9*A–C’*). In either form, occurrence of α-syn immunoreactivity in these areas did not appear to result in disrupted morphology or architecture of local neuronal populations. Of note, cresyl violet staining of entorhinal cortex, hippocampus, and piriform cortex revealed no evidences of neurodegeneration and/or neuronal loss in α-syn-injected rats (Fig. 9*J–L*; compare with corresponding regions in control animals, Fig. 9*G–I*). Moreover, separate morphometric analyses, conducted to assess the possible toxic effects of α-syn overexpression, revealed that at no time point postinjection was any obvious loss of dopaminergic, cholinergic, or noradrenergic neurons detected in the substantia nigra (SN), the ventral tegmental area (VTA), medial septum/diagonal band of Broca (MS/DBB), or locus coeruleus (LC), respectively. Thus, occurrence and distribution of tyrosine hydroxylase (TH)-immunoreactive, choline acetyltransferase (ChAT)-immunoreactive, or dopamine-β-hydroxylase (DBH)-immunoreactive neurons did not differ between α-syn-injected and control animals in any of the analyzed regions (data not shown).

**Figure 10.**
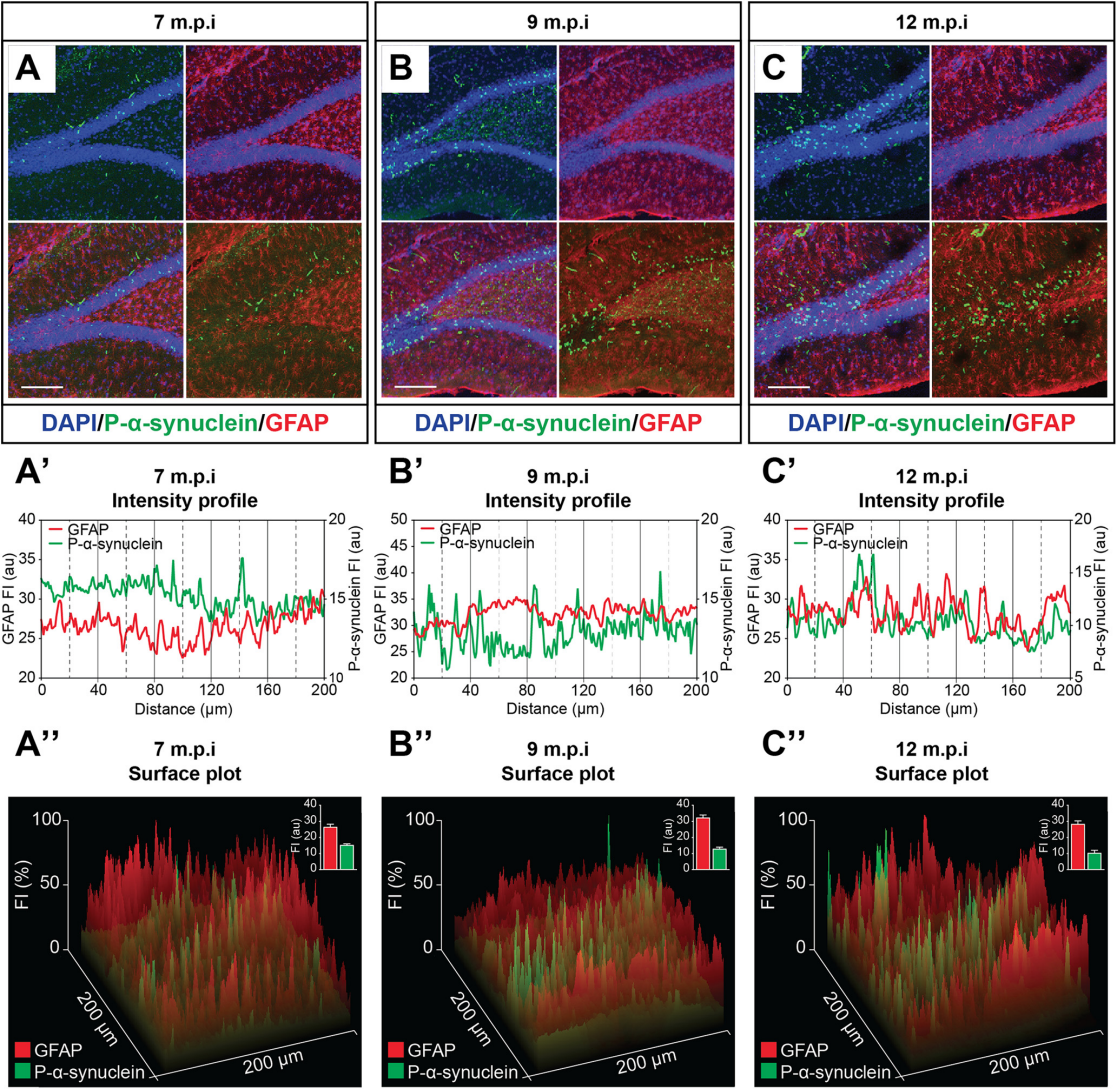
Confocal microscopy images illustrating the progressive increase of hippocampal Ser129-phosphorylated α-syn (***A–C***) and the lack of co-localization with GFAP at seven, nine, and 12 months after the injection of recombinant mouse α-syn fibrils into the hippocampus. In the lower diagrams, analysis of fluorescence intensity (***A’–C’***) and surface plot profiles (***A”–C”***) of confocal images in ***A–C*** indicate very poor or no glial occurrence of the Ser129-phosphorylated α-syn. Scale bars: 50 μm (***A–C***).

Consistent with the above observations of a time-dependent increase of PK-resistant α-syn aggregates within the hippocampus, immunofluorescence confirmed the progressive increase of Ser129-phosphorylated α-syn in the hippocampus of injected rats (Fig. 10*A–C*), whereas no such immunoreactivity was detected in controls (data not shown). Subsequent analyses of fluorescence intensity and localization profile in P-α-syn-GFAP double immunostained specimens at seven, nine, and 12 months postinjection revealed virtually no co-localization of these markers. In fact, close inspection of the expression profile plots of P-α-syn and GFAP immunoreactivities showed very poor or no juxtaposition between the respective peaks, thus ruling out a glial occurrence of Ser129-phosphorylated α-syn as a result of intracerebral α-syn fibril treatment at any time point postinjection (Fig. 10*A’–C’*, *A”–C”*).

### Long-term effects of α-syn fibril injection

#### Western blot analyses

**Figure 11.**
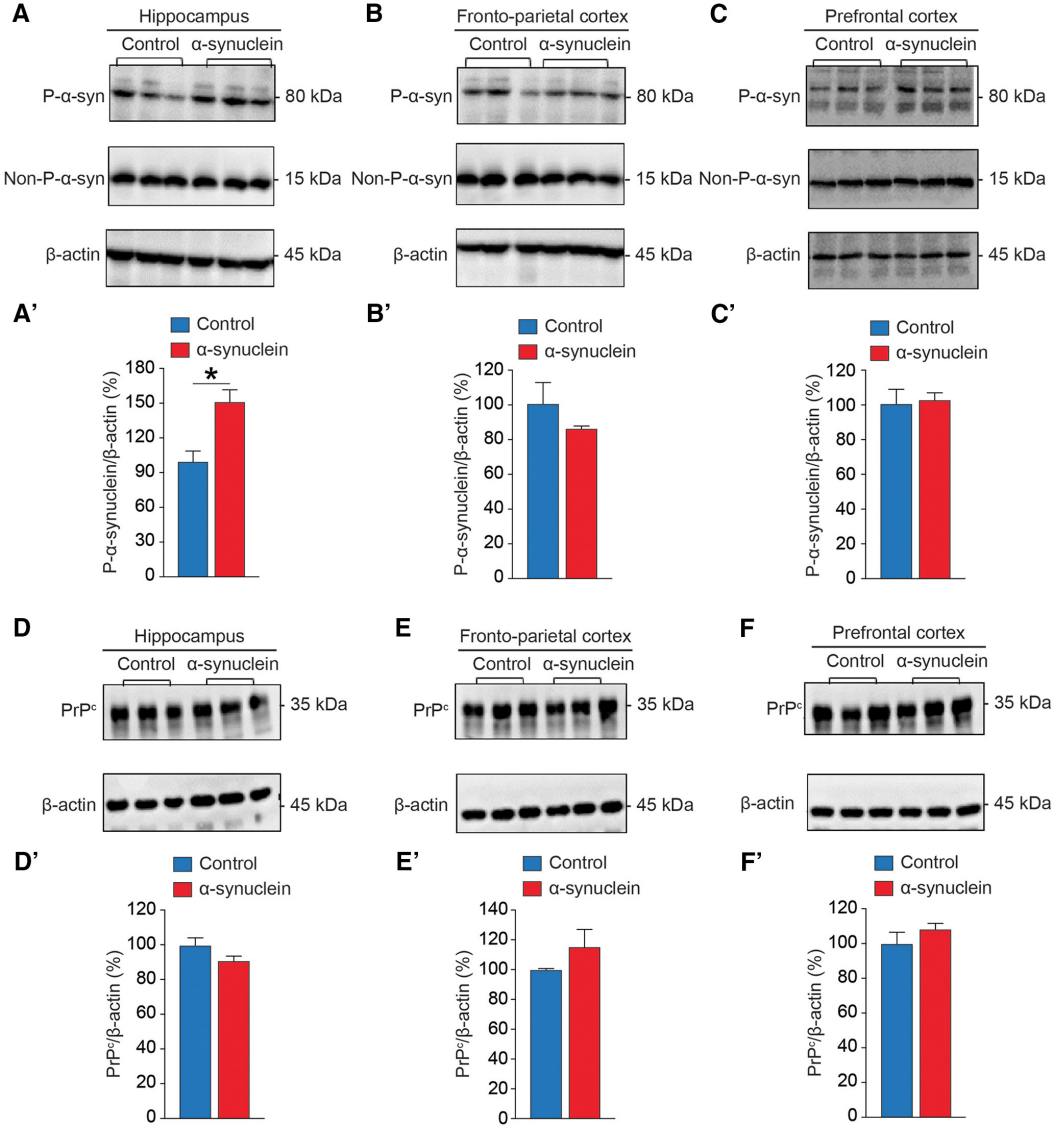
Long-term effects of intrahippocampal α-syn fibril injection on regional tissue levels of Ser129-phosphorylated α-syn (P-α-syn), non-phosphorylated α-syn (non-P-α-syn), and PrP^c^. Representative Western blotting bands and densitometric analyses illustrate the expression levels of the various proteins in the hippocampus (***A***, ***A’***, ***D***, ***D’***), fronto-parietal cortex (***B***, ***B’***, ***E***, ***E’***), and prefrontal cortex (***C***, ***C’***, ***F***, ***F’***) of rats injected with α-syn fibrils and controls at 12 months postinjection. The values are shown as a percentage of P-α-syn, non-P-α-syn forms, or PrP^c^ relative to β-actin, this latter being the loading control. Data are represented as mean ± SEM. Asterisk in ***A’*** indicates significant difference from control at *p* < 0.05.

At 12 months postinjection, Western blot analyses were conducted on tissue homogenates from the hippocampus and the prefrontal and frontoparietal cortices of control and α-syn-injected rats. Because of technical reasons (accidental loss of the homogenate samples), Western blot analyses on the entorhinal cortex could not be conducted and will not be reported here. The analyses were designed to obtain estimates of the relative levels of non-phosphorylated and Ser129-phosphorylated α-syn. The procedure (as described above) enabled the detection of a ∼15-kDa band, which represents the non-phosphorylated α-syn (Fujiwara et al., 2002), a ∼80-kDa which correspond to Ser129-phosphorylated α-syn (Fig. 11*A–C*), and a ∼35-kDa for PrP^C^ (Fig. 11*D–F*). Densitometric analysis of the bands after normalization against β-actin revealed a significant 50% increase of the Ser129-phosphorylated α-syn level only in the hippocampus of α-syn-treated rats compared with controls (*p* < 0.01; Fig. 11*A* ***’***). By contrast, no group differences in Ser129-phosphorylated α-syn expression were detected in prefrontal and frontoparietal cortices (Fig. 11*B’–C’*), nor did the α-syn treatment affect the expression of non-phosphorylated α-syn in any of the analyzed regions. Likewise, the expression levels of PrP^C^ in these regions were similar in the two groups (Fig. 11*D’–F’*).

#### qRT-PCR analysis

**Figure 12.**
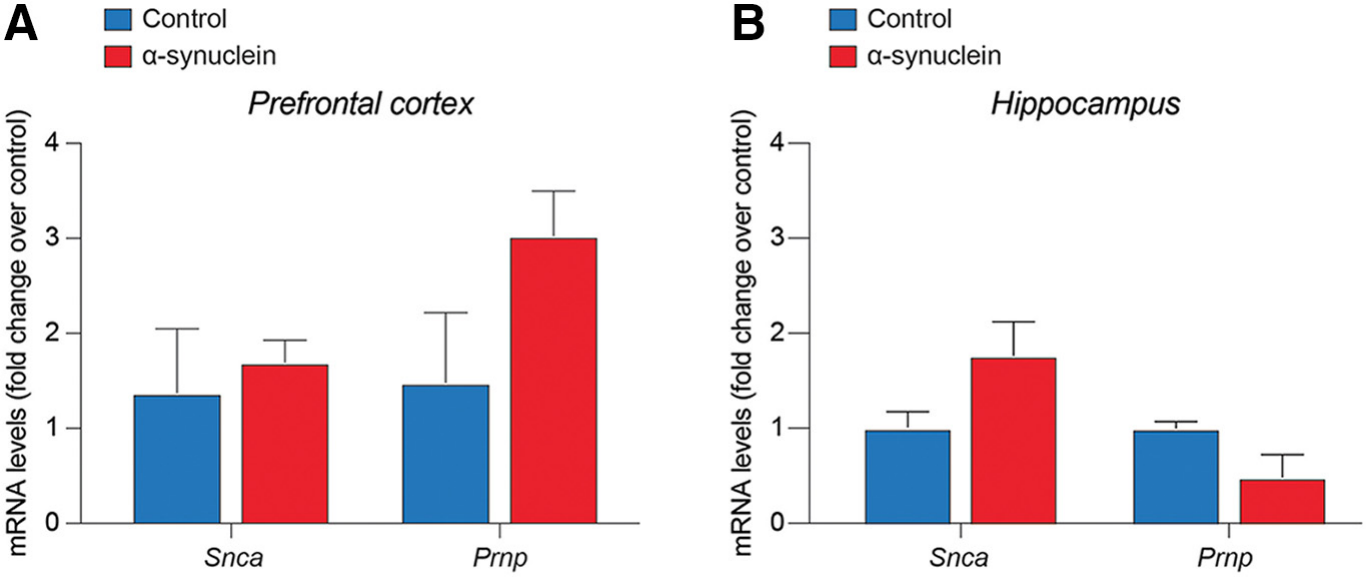
Quantification of the mRNA expression levels of *Snca* and *Prnp* in prefrontal cortex (***A***) and in the hippocampus (***B***) of rats injected with α-syn fibrils and controls at 12 months postinjection. Data are represented as mean fold change over control ± SEM.

To evaluate the differential gene expression of *Scna* and *Prnp*, qRT-PCR analysis was performed on tissue homogenates from prefrontal cortex and hippocampus of control and α-syn-injected rats at 12 months postinjection. Consistently, no significant changes in the expression level of *Scna* (encoding for α-syn) and *Prnp* (encoding for PrP^C^) mRNA were detected in either the prefrontal cortex (Fig. 12*A*) or the hippocampus (Fig. 12*B*) of α-syn-injected rats as compared with control.

## Discussion

The aim of this study was to evaluate the anatomic, neurochemical, molecular, and behavioral effects of recombinant mouse α-syn PFF following intrahippocampal injection in the adult rat. The injections resulted in the time-dependent occurrence of PK-resistant α-syn aggregates within the hippocampus, suggesting that neurons in this region may be particularly susceptible to transmitted α-syn pathology. Moreover, in keeping with previous findings following intrastriatal/intracortical administration in mice (Luk et al., 2012), the present data lend support to the notion that pathologic α-syn can spread over considerable distances to several central regions. In fact, PK-resistant α-syn aggregates were also found in anatomically related areas, such as the EC, the vDBB, and the piriform cortices, which, interestingly, was associated to the onset of specific deficits in spatial working memory, i.e., the memory function that is commonly impaired in PD patients (Bradley et al., 1989).

Thus, at seven months postinjection, α-syn immunoreactivity in the hippocampus was mainly associated to neuritic processes, and relatively few intracellular inclusions were detected. In the hippocampus, the numbers of Lewy-like neuritic inclusions did not vary significantly over time, whereas in the EC, the estimated numbers of α-syn-immunoreactive neurites were seen to increase between seven and nine months but remained virtually unchanged thereafter. In both regions, the immunoreactive material appeared to progressively invade somata, and already at nine months, the number of neuronal profiles with dense perinuclear LB-like inclusions was seen significantly increased. Interestingly, inclusions of α-syn-immunoreactive material in both the hippocampus and EC exhibited the most dramatic increases (by ∼6-fold and 10-fold, respectively) at 12 months postinjection. The time course and dynamics of intracellular α-syn occurrence, with more dramatic increases detected between nine and 12 months postinjection, i.e., concurrently to the onset of cognitive impairments, points to a precise time window, relatively long after its injection into the brain parenchyma, when α-syn is capable to massively accumulate inside neurons and efficiently interfere with their function. The present findings appear consistent with previous observations of remarkably slow progression of α-syn pathology in PD patients (Halliday et al., 2008; Kim et al., 2014), thus confirming the feasibility of the α-syn PFF infusion paradigm as a tool for modeling aspects of cognitive loss n PD. In fact, a significant trend toward a worsened performance in the RAWM task was seen from nine months postinjection, to become dramatically evident at 12 months. Interestingly, at no time point postinjection was any motor disturbance detected in the injected animals, nor was α-syn found within regions rich in dopamine neurons and fibers, such as the SN, the VTA or the striatum. In addition, no obvious loss in dopaminergic, noradrenergic, or cholinergic immunoreactivity was detected in α-syn-injected animals compared with controls, thus the behavioral impairments seen in the α-syn-injected animals appear to be determined by the long-term persisting α-syn neuropathology in the affected neurons rather than by neurodegeneration, which is consistent with the observations by Hall et al. (2013). These observations are also in keeping with the findings of a recent *in vitro* study (Froula et al., 2018) showing that treatment of excitatory hippocampal neurons with α-syn fibrils induced neuronal dysfunctions, such as a reduced frequency and amplitudes of spontaneous Ca^2+^ transients (Overk et al., 2015), as well as a dramatic disruption of synaptic activity, and accelerated neuronal death (Wu et al., 2019). In particular, the reduced spine density induced by α-synucleinopathy may be a pathophysiological phenotype contributing to dementia (Blumenstock et al., 2017; Kramer and Schulz-Schaeffer, 2007). Froula and colleagues also found some compensatory mechanisms responding to synaptic defects induced by α-syn fibrils, which could explain the long-time window before the onset of memory impairments (Froula et al., 2018).

Considering the occurrence of α-syn immunoreactivity in the entorhinal cortex and, at 12 months postinjection, in the vDBB and the piriform cortices, i.e., all regions involved in memory functions and the time course and regional pattern of α-syn accumulation, the present data therefore suggest that hippocampal Lewy pathology must spread beyond its initial site, toward cognitively-relevant areas, to induce measurable memory dysfunctions. This is consistent with the model proposed by Adamowicz et al. (2017), according to which learning, and memory, impairment become apparent once LB pathology concurrently affects CA1 and entorhinal cortex.

The mechanism underlying such spreading needs to be further elucidated. However, it has been reported that deficits in learning and memory observed in α-syn transgenic mice are accompanied by alterations in postsynaptic densities (Masliah et al., 2011) or reduction in presynaptic vesicle-associated proteins such as synaptophysin (Lim et al., 2011), suggesting that the toxicity of α-syn may be mediated by disturbances in synaptic transmission (Larsen et al., 2006; Nemani et al., 2010). Here, we did not find any colocalization of synapsin-1 immunoreactivity in α-syn-positive hippocampal neurons (data not shown), which nevertheless does not exclude such possibility and warrants further analyses.

In a recent investigation, Aulić et al. (2017) found that the PrP^C^ binds α-syn amyloids to get access into the cell and accumulate into cytoplasm, thereby facilitating the cell-to-cell spreading of the pathology. Likewise, it has been reported that α-syn oligomeric species interact with PrP^C^ through mGluR5, activating SFK kinases and, subsequently, NMDAR2B to induce neuronal dysfunction and cognitive deficits in Thy1-aSyn mice and also that the toxic effects induced by α-syn oligomers may be prevented using antibody-mediated inactivation of PrP^C^ (Ferreira et al., 2017). Taken together, these studies have identified previously unknown components of the signaling cascade triggered by α-syn, therefore suggesting that PrP^C^ signaling might be involved in the pathogenesis of synucleinopathies and may be used as a potential therapeutic target to prevent or slow down the progression of synucleinopathies. Considering that the α-syn-PrP^C^ interactions reported by both Aulić et al. (2017) and Ferreira et al. (2017) are detected in five- to eight-month-old animals, it is possible to assume the existence of specific stages during which the interaction of these proteins may actually take place, leading to a massive α-syn spreading, to become less pronounced at later time points, once the spreading has occurred. This may be of potential interest, as it may highlight a valuable window of therapeutic opportunity, whose full understanding, again, requires further investigation.

PrP^C^ levels in the hippocampus, prefrontal and fronto-parietal cortices of α-syn-injected rats was similar to controls at 12 months postinjection, as well as *Prnp* mRNA and *Snca* mRNA levels in the prefrontal cortex which is consistent with previous observations (Aulić et al., 2017). This finding appears to confirm that the level of PrP^C^ and α-syn transcripts were not altered after fibrils injection and that the observed increase of pathologic α-syn is likely to occur at protein level.

Increased level of phosphorylated α-syn at Ser129 represents one of the hallmarks of PD. In fact, ∼90% of α-syn deposited in LBs is phosphorylated at Ser129 while only 4% or less of the total α-syn is phosphorylated at this residue in brains from individuals without PD (Fujiwara et al., 2002; Anderson et al., 2006). Nevertheless, the significance of phosphorylation in the biology and pathophysiology of the protein is still controversial, and both *in vitro* and *in vivo* studies, examining phosphorylation of α-syn at different sites, have reported conflicting results, showing either promotion (Smith et al., 2005), inhibition (Paleologou et al., 2008), or no effect (Fiske et al., 2011) on inclusion formation. However, to assess the role, if any, of the phosphorylation of α-syn fibrils in the seeding and pathologic accumulation of α-syn, it was found that P-PFF are more efficiently uptaken by neurons after injection in wild-type mice, leading to increased seeding and accumulation of the endogenous α-syn (Karampetsou et al., 2017). This evidence suggests that phosphorylation may promote aggregation to the α-syn assemblies and as such may regulate the onset of neuronal dysfunction. Similarly, we found after injection of non-phosphorylated recombinant mouse α-syn fibrils in rats, a time-dependent increase of Ser129-phosphorylated α-syn into the hippocampus with the overt manifestation of cognitive deficits. Our data, therefore, not only provide a further proof of the importance of phosphorylation in the pathogenesis of disease but also allow the validation of our experimental model.

In conclusion, our study shows that bilateral injection of recombinant α-syn PFF into the hippocampus of rat is sufficient to trigger α-syn pathology within the hippocampus, which spreads to entorhinal cortex, vDBB, and piriform cortex, and leads to working memory impairment. Although the mechanism behind α-syn-mediated working memory impairment has to be further elucidated, the present study provides an additional proof that α-syn may be one the main neuronal substrates for cognitive impairment in synucleinopathies.

## Synthesis

Reviewing Editor: Julie Andersen, Buck Institute Decisions are customarily a result of the Reviewing Editor and the peer reviewers coming together and discussing their recommendations until a consensus is reached. When revisions are invited, a fact-based synthesis statement explaining their decision and outlining what is needed to prepare a revision will be listed below. The following reviewer(s) agreed to reveal their identity: Hossein Aleyasin. Note: If this manuscript was transferred from JNeurosci and a decision was made to accept the manuscript without peer review, a brief statement to this effect will instead be what is listed below.

This manuscript provides valuable data towards a better understanding of the role of a-Syn aggregation in the formation of Lewy bodies and Lewy neurites in non-dopaminergic neurons in areas of the forebrain that affect cognitive function. As such, it extends previous observations of anatomical and neuronal substrates underlying cognitive impairment in PD.

The manuscript is logically written and provides a detailed description of anatomical distribution, molecular characterization, and functional outcome of bilateral intrahippocampal injection of preformed fibrillary (PFF) alpha-synuclein over the course of 12 months in a rat model. The study is descriptive and correlative by design and does not address mechanisms that are involved in the processes of aggregation, propagation and phosphorylation of alpha-synuclein and ultimately formation of Lewy bodies and Lewy neurites (LB/LN). However, the findings rule out the necessity of exploring some proposed mechanisms such as interaction with cellular prion protein (PrPC) or increase transcription of alpha-synuclein. The data included in the manuscript sufficiently supports the authors’ conclusions. There are a few remaining questions/comments however that need to be addressed:

It is clearly mentioned in the manuscript that long-term effects of alpha-synuclein are associated with “neuropathology rather than neurodegeneration", but all areas that have been evaluated for neurodegeneration are located in the brainstem (SN,LC, and VTA). Does aggregation of alpha-synuclein result in degenerative changes, including neuronal death, in affected areas such as the hippocampus, entorhinal, and piriform cortex? Without presenting this data, it is hard to support the conclusions regarding affected areas.

Presentation of the negative data demonstrating lack of change in Prnp or a-Syn mRNA in hippocampus tissue samples from alpha-synuclein injected versus control samples is essential in order to demonstrate that increased aggregation of a-synuclein and formation of LB occurs by a mechanism not involving overexpression of a-Syn.

What is the rationale for choosing only female rats?

Extended data that are provided are integral to the study and could be easily added to the main figures.
