# Supplementary figures and images for: Chronic α-Synuclein Accumulation in Rat Hippocampus Induces Lewy Bodies Formation and Specific Cognitive Impairments
Source: eNeuro. 2020 Jun 15;7(3):ENEURO.0009-20.2020. doi: 10.1523/ENEURO.0009-20.2020 (PMC7307628; doi:10.1523/ENEURO.0009-20.2020)

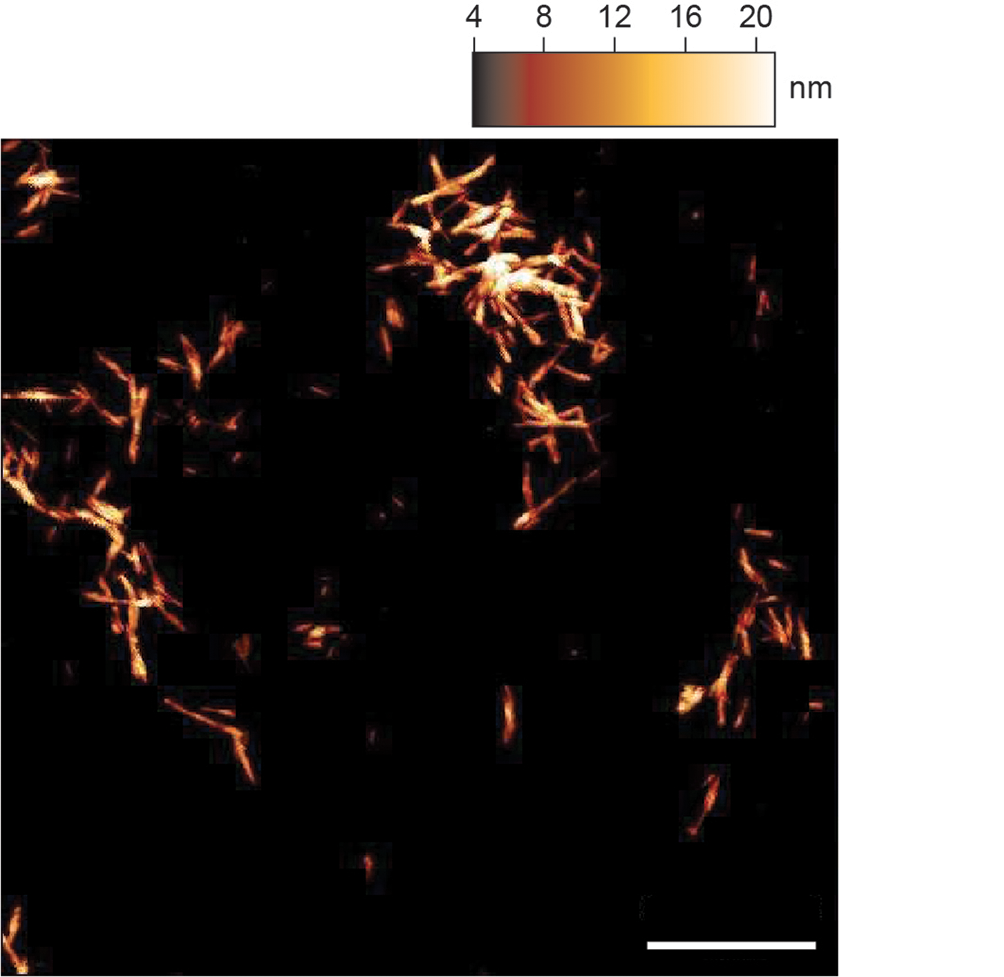

Supplement: Extended Data Figure 1-1 — AFM micrograph showing the assemblies of the pre-aggregated α-syn fibrils prior to their intrahippocampal inoculation. Scale bar: 1 μm. Download Figure 1-1, TIF file [file enu-eN-NWR-0009-20-s01.tif]
